# Roles of *rpoS*-activating small RNAs in pathways leading to acid resistance of *Escherichia coli*

**DOI:** 10.1002/mbo3.143

**Published:** 2013-12-08

**Authors:** Geunu Bak, Kook Han, Daun Kim, Younghoon Lee

**Affiliations:** Department of Chemistry, KAISTDaejeon, 305-701, Korea

**Keywords:** Acid resistance, *Escherichia coli*, RpoS, small noncoding RNA.

## Abstract

*Escherichia coli* and related enteric bacteria can survive under extreme acid stress condition at least for several hours. RpoS is a key factor for acid stress management in many enterobacteria. Although three *rpoS-*activating sRNAs, DsrA, RprA, and ArcZ, have been identified in *E. coli*, it remains unclear how these small RNA molecules participate in pathways leading to acid resistance (AR). Here, we showed that overexpression of ArcZ, DsrA, or RprA enhances AR in a RpoS-dependent manner. Mutant strains with deletion of any of three sRNA genes showed lowered AR, and deleting all three sRNA genes led to more severe defects in protecting against acid stress. Overexpression of any of the three sRNAs fully rescued the acid tolerance defects of the mutant strain lacking all three genes, suggesting that all three sRNAs perform the same function in activating RpoS required for AR. Notably, acid stress led to the induction of DsrA and RprA but not ArcZ.

## Introduction

Most pathogenic or nonpathogenic enterobacteria grow optimally at a pH range 5–9, and are therefore classified as neutrophilic bacteria. However, bacteria often face drastic pH changes along the gastrointestinal tract, such as gastric acid (pH 1.5 ˜ 2.5), alkaline secretion of pancreas (pH ˜ 9), and weak organic acid metabolites in intestine. The ability of enterobacteria to survive these conditions is critical for their colonization in host intestine or pathogenicity.

*Escherichia coli* can survive in extreme acidic condition (pH < 2.5) at least for several hours, and its acid resistance (AR) systems have thus been extensively studied (Lin et al. 1996). At least three discernible AR systems are responsible for protecting *E. coli* cells from acid stress (Foster 2004; Zhao and Houry 2010). Induction of each AR system varies depending on environmental conditions, such as growth/challenge media, growth phase, aerobic/anaerobic conditions, challenging pH, or exogenous amino acids. The AR1 system has been identified in cells grown oxidatively in rich media to the stationary phase, and shown to require *rpoS* and *crp* (Small et al. 1994; Castanie-Cornet et al. 1999). The glutamate-dependent AR2 and arginine-dependent AR3 systems include amino acid–dependent decarboxylase complexes comprising amino acid decarboxylases and countertransporters that exchange decarboxylation products for new extracellular amino acids. Recently, another AR system relying on L-glutamine, GadC, and glutaminase that releases gaseous ammonia to neutralize protons was reported (Lu et al. 2013).

The glutamate-dependent AR2 system plays a major role in AR and is most effective in relieving acid stress at very low pH (<2.5). The AR2 system contains two glutamate decarboxylases (GadA and GadB), and its countertransporter (GadC), which exchange *γ*-amino butyric acid (the decarboxylation product of glutamate) for new external glutamate (De Biase et al. 1999; Ma et al. 2012). Expression of *gadA* and *gadBC* operon is stimulated by the key activator, GadE (Ma et al. 2003a; Castanie-Cornet et al. 2010), and GadX activates *gadE* expression (Sayed et al. 2007; Sayed and Foster 2009). Transcription of *gadX* is primarily induced by the alternative sigma factor, RpoS (Tramonti et al. 2002). cAMP receptor protein (CRP) affects the AR2 system by repressing RpoS transcription (Ma et al. 2002, 2003b). Histone-like protein, H-NS, has been shown to directly suppress *gadA* and *gadBC* expression (Waterman and Small 2003; Giangrossi et al. 2005). Two-component systems, EvgA/S (Ma et al. 2004), PhoP/Q (Zwir et al. 2005), and TorS/R (Bordi et al. 2003), additionally regulate *gadE* transcription.

It is also known that three Hfq-interacting small noncoding RNAs, GadY, DsrA, and GcvB, participate in conferring AR to *E. coli* cells. GadY is encoded on the antisense strand between *gadX* and *gadW*, and positively regulates these genes by affecting processing of *gadXW* transcripts by RNase III (Opdyke et al. 2004, 2011; Tramonti et al. 2008). Overexpression of DsrA renders *E. coli* cells resistant to acid stress by inducing AR-related genes, such as *hdeAB*, *gadAX*, and *gadBC* (Lease et al. 2004), possibly through the actions of RpoS and/or H-NS, as DsrA is known to enhance *rpoS* translation and *hns* mRNA turnover (Sledjeski and Gottesman 1995; Sledjeski et al. 1996; Majdalani et al. 1998; Lease and Belfort 2000). GcvB, a regulator of genes involved in amino acid metabolism (Urbanowski et al. 2000; Pulvermacher et al. 2009a,2009b), enhances AR in a *rpoS*-dependent manner (Jin et al. 2009), although the underlying mechanism remains elusive.

In addition to AR, RpoS is crucial for general stress management and virulence in many pathogenic or nonpathogenic enterobacteria (Dong and Schellhorn 2010; Battesti et al. 2011). Three Hfq-interacting sRNAs, DsrA, RprA, and ArcZ, were reported to positively regulate *rpoS* expression (Sledjeski et al. 1996; Majdalani et al. 1998, 2001; Mandin and Gottesman 2010). Therefore, it is not difficult to speculate that these sRNAs would provide *E. coli* with AR by activating *rpoS* expression, but roles of these small RNA molecules in pathways leading to AR remain elusive due to the complexity of mechanisms of AR.

In this study, we showed the acquisition mechanisms of AR by *E. coli* cells through *rpoS*-activating sRNAs. The acquisition depends on RpoS and partially on GadX. Interestingly, DsrA and RprA, but not ArcZ, act as mediator molecules for the acid stress response. Our data should aid in improving our current understanding of the acquisition mechanisms of AR by *E. coli* cells through *rpoS*-activating sRNAs.

## Experimental Procedures

### Strains and plasmids

The bacterial strains and plasmids used in this study are listed in Table1. MG1655 deletion mutants of *rpoS*, *hfq*, *arcB*, *gadX*, or *gadW* were obtained by P1 transduction using the deletion strains from the *E. coli* Keio strain collection (Baba et al. 2006). Knockout of sRNA genes was performed as described previously (Datsenko and Wanner 2000), using *λ*Red-mediated recombination with pKD46 plasmid and kanamycin cassettes generated with polymerase chain reaction (PCR) using pKD13 as a template plasmid and the oligonucleotides listed in Supplementary Table 1. In case of the *arcZ* mutant, the-10 promoter mutation of *arcZ* was introduced to avoid unexpected effects on expression of the overlapped *arcB* gene because the kanamycin cassette sequence could be overlapped if the *arcZ* structural sequence is replaced with the kanamycin cassette. The kanamycin cassette for the *arcZ* mutant was generated as follows. A DNA fragment containing the region of positions −230 to −191 with respect to the transcription start of *arcZ* followed by the kanamycin resistance gene was prepared by PCR amplification using a ΔP10arcZP1/ΔP10arcZP2 primer pair. Another fragment carrying the −150 to +48 region of *arcZ* was prepared by PCR amplification using a ΔP10arcZP3/ΔP10arcZP4 primer pair. The two amplified PCR products were then linked using overlap extension PCR (Horton et al. 1989) with ΔP10arcZP1/ΔP10arcZP4 primer pair. This cassette was cloned into a pGEM-T vector (Promega, Tokyo, Japan), and site-directed mutagenesis of the −10 promoter region was performed. The mutated cassette containing the mutated-10 sequence (CTCGAG) was amplified using PCR. Kanamycin marked mutations were transferred into a desired strain background using bacteriophage P1 transduction (Silhavy et al. 1984). No expression of ArcZ in the *arcZ* mutant was confirmed by Northern blot (Fig. S4). To obtain MG1655*Δ*3 with deletion of all three *rpoS*-activating sRNA genes, the Flp recognition target (FRT)-flanked kanamycin cassette introduced into the first *dsrA* deletion strain was removed using Flp recombinase from pCP20 plasmid (Cherepanov and Wackernagel 1995), an additional *rprA* deletion was introduced by P1 transduction, the kanamycin cassette was removed again, and the *arcZ* promoter mutation was finally introduced by P1 transduction.

**Table 1:** Strains and plasmids used in this study.

Name	Description	Source
Strains		
MG1655	*E. coli* MG1655 wild type	Laboratory stock
*ΔarcZ*	MG1655 *ΔarcZ::kan^*R*^* (promoter-10 mutant)	This study
*ΔdsrA*	MG1655 *ΔdsrA::kan*^*R*^	This study
*ΔrprA*	MG1655 *ΔrprA::kan*^*R*^	This study
MG1655*Δ*3	MG1655 *ΔdsrA ΔrprA ΔarcZ::kan*^*R*^	This study
BW25113*ΔrpoS*	*E. coli* BW25113 *ΔrpoS::kan*^*R*^	Keio collection (Baba et al. 2006)
BW25113*ΔarcB*	*E. coli* BW25113 *ΔarcB::kan*^*R*^	Keio collection (Baba et al. 2006)
BW25113*ΔgadX*	*E. coli* BW25113 *ΔgadX::kan*^*R*^	Keio collection (Baba et al. 2006)
BW25113*ΔgadW*	*E. coli* BW25113 *ΔgadW::kan*^*R*^	Keio collection (Baba et al. 2006)
BW25113*Δhfq*	*E. coli* BW25113 *Δhfq::kan*^*R*^	Keio collection (Baba et al. 2006)
*ΔrpoS*	*E. coli* MG1655 *ΔrpoS::kan*^*R*^	This study
*ΔarcB*	*E. coli* MG1655 *ΔarcB::kan*^*R*^	This study
*ΔgadX*	*E. coli* MG1655 *ΔgadX::kan*^*R*^	This study
*ΔgadW*	*E. coli* MG1655 *ΔgadW::kan*^*R*^	This study
*Δhfq*	*E. coli* MG1655 *Δhfq::kan*^*R*^	This study
SG30013	MG1655 *lacX74*_att_RpoS_750_::LacZ, Reporter for transcription, translation, and degradation of RpoS	S. Gottesman (Wassarman et al. 2001)
Plasmids		
pHMB1	A derivative of pHM1 (Han et al. 2010), Amp^R^, IPTG-inducible transcription from immediate after *Eco*RI site, modified *rnpB* terminator (GAUUU to GGAGU) next to *Xba*I site.	This study
pArcZ	pHMB1 carrying *arcZ*	This study
pDsrA	pHMB1 carrying *dsrA*	This study
pRprA	pHMB1 carrying *rprA*	This study
pGEM3	Cloning vector, Amp^R^	Promega
pGEM-T	Cloning vector, Amp^R^, Site-directed mutagenesis for *arcZ*	Promega
pG-ArcZ	pGEM3 carrying *arcZ*	This study
pBAD-myc-hisC	Control vector for pBAD-RpoS plasmid	Invitrogen
pBAD-RpoS	*rpoS* cloned in pBAD24, Amp^R^	Zhou and Gottesman (2006)
pKD46	*λ*Red recombination system under control of arabinose-inducible promoter, temperature-sensitive replication, Amp^R^	Datsenko and Wanner (2000)
pKD13	Template plasmid for FRT-flanked kanamycin resistance cassette.	Datsenko and Wanner (2000)
pCP20	FLP^+^, *λ* cI857^+^, *λ* P_R_ Rep^ts^, Amp^R^, Cm^R^, expression of site-specific Flp recombinase under control of heat-inducible promoter, temperature-sensitive replication.	Cherepanov and Wackernagel (1995)

pG-ArcZ was constructed using the PCR-amplified DNA fragment containing the *arcZ* gene sequence from −107 to +208 relative to the transcription start site of ArcZ. For isopropyl β-D-1-thiogalactopyranoside (IPTG)-inducible overexpression of each sRNA, the pHMB1 plasmid was constructed by replacing the *Aat*II site of pHM1 (Han et al. 2010) with a *Xba*I site and adding the modified *rnpB* terminator sequence (Kim et al. 1996) with mutations at the *rne-*dependent cleavage site (from GAUUU to GGAGU) adjacent to the *Xba*I site. ArcZ, DsrA, and RprA genes were amplified from MG1655 genomic DNA using PCR, and cloned into *Eco*RI and *Xba*I sites of pHMB1. The resulting plasmids express sRNAs from their native transcription start site and terminate transcription at the native terminator of each sRNA gene.

### Protein identification

*E. coli* MG1655 cells containing control vector pGEM3 or pG-ArcZ were cultured overnight, diluted 1:100 into fresh lysogeny broth (LB) media, and grown for 17 h at 37°C. Cells were harvested, resuspended in phosphate buffered saline (PBS) buffer, and sonicated. After sonication, supernatant fractions were electrophoresed on a 10% sodiumdodecyl sulfate-polyacrylamide gel (SDS-PAGE). Protein bands of interest were isolated and digested in the gel with trypsin. Proteins were analyzed by electrospray hybrid quadrupole-time-of-flight tandem mass spectrometer (Q-TOF2 MS/MS).

### RNA extraction and Northern blot analysis

Cells were grown overnight in LB broth with 100 *μ*g mL^−1^ ampicillin if necessary. Overnight cultures were diluted 1:100 in fresh media and growth continued. For overexpression of sRNA, 1 mmol/L IPTG was added to cell cultures grown for 1 h after 1:100 dilution. Total cellular RNAs were extracted from culture at the desired time points by acidic hot phenol method, as described previously (Kim et al. 1996).

Northern blot analysis was carried out as described (Tezias et al. 2012). Briefly, 10 *μ*g of total RNAs were fractionated on 7 mol/L urea, 5% PAGE, and electrotransferred onto a Hybond™-XL membrane (Amersham Biosciences, Little Chalfont, UK). Membranes were hybridized with ^32^P-labeled DNA probes in Rapid-Hyb buffer (Amersham Biosciences) according to the manufacturer's instructions. Hybridization signals were analyzed by using Image Analyzer FLA7000 (Fuji, Tokyo, Japan).

### AR **assay**

Three colonies for each strain were pooled in LB. The overnight culture was diluted 1:100 in fresh LB and grown at 37°C. Exponentially growing cells were obtained from cultures grown for 2.5 h and stationary-phase cells from cultures grown for 12 h after dilution, respectively. If necessary, LB media containing 100 *μ*g mL^−1^ ampicillin was used, and 1 mmol/L IPTG was added to diluted cultures grown for 1 h.

To assay AR, sample cells were harvested and resuspended M9 minimal media supplemented with 0.4% glucose. The suspended sample (˜10^8^ cells mL^−1^ for exponential phase, ˜10^9 ^cells mL^−1^ for stationary phase) was diluted 1:10 or 1:100 with M9 minimal media supplemented with 0.4% glucose and 1.5 mmol/L glutamate, which was previously adjusted to pH 2.0 or 3.0 with concentrated HCl and incubated for 1 h at 37°C in a shaking incubator. Cells in 2 mL medium in a 14 mL test tube were grown with shaking at 250 rpm. pH changes were not observed during acid challenge of 1 h.

After acid exposure, cells were serially diluted in LB and plated on LB agar plates. The number of viable colonies was determined to calculate the colony-forming units (CFU mL^−1^). The survival rate was calculated based on the ratio of CFU of cells after acid exposure to CFU before the acid exposure. In parallel, cultures exposed to acid challenge were additionally diluted 1:10 serially in LB and 10 *μ*L of each dilution was spotted onto LB plates.

### *β*-galactosidase assay

Three colonies for each strain were pooled in LB, and the overnight culture was diluted 100-fold in fresh LB media–containing ampicillin (100 *μ*g mL^−1^) and 1 mmol/L IPTG, if necessary. At the desired time points, the *lacZ* fusion reporter gene was assayed as described previously (Zhang and Bremer 1995; Lee et al. 2013). At least three independent experiments were performed.

## Results

### Overexpression of ArcZ leads to GadA accumulation in E. coli

ArcZ is a highly conserved sRNA in bacteria that was initially identified using genome-wide sRNA screening by independent research groups (Argaman et al. 2001; Wassarman et al. 2001). This sRNA is highly associated with Hfq protein in *E. coli* and *Salmonella* (Zhang et al. 2003; Sittka et al. 2008). Papenfort et al. (2009) characterized the function of ArcZ sRNA as a negative regulator of several mRNAs using microarray in *Salmonella*, whereas Mandin and Gottesman (2010) showed that ArcZ sRNA acts as a positive regulator of *rpoS* as well as a negative regulator of *arcB* in *E. coli*.

**Figure 1:**
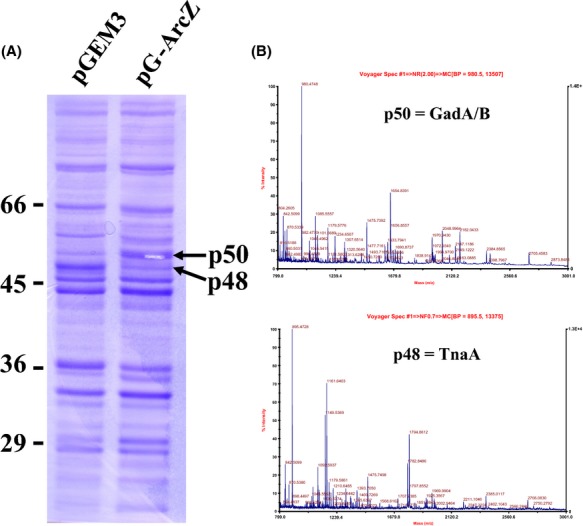
Protein expression profile of ArcZ overexpressing cells. (A) Equal amounts of total protein from *Escherichia coli* MG1655 cells containing pG-ArcZ and the pGEM3 vector, respectively, were subjected to electrophoresis on a 10% acrylamide SDS gel and compared. Increased or decreased protein bands are indicated with arrows. (B) Protein bands were excised and identified using Q-TOF2 MS/MS. As peptides containing the N-terminal end regions of p50 were not detected by Q-TOF MS/MS, GadA and GadB were indistinguishable so that p50 was assigned to GadA or GadB (GadA/GadB).

As an independent study of ArcZ function in *E. coli*, we initially examined the changes in protein expression patterns upon overexpression from multiple copies of *arcZ* on a multicopy plasmid. Bands displaying highly increased or decreased intensity were excised and identified by Q-TOF2 MS/MS. GadA/B, glutamate decarboxylases of the AR2 system, was highly induced, whereas the amount of TnaA, an alkali-inducible tryptophanase (Bordi et al. 2003), was significantly reduced (Fig. 1). This reciprocal change in the protein profile is similar to the case of GadY-overexpressed cells previously reported (Opdyke et al. 2004). GadY-overexpressed cells showed increased DnaK, GroEL, and GadA levels, and decreased TnaA expression. As a sRNA, GadY, positively regulates GadX, a transcriptional activator of *gadA* and *gadBC* operons, via base pairing with the 3′-UTR of *gadX* mRNA.

### Effects of ArcZ on AR

**Figure 2:**
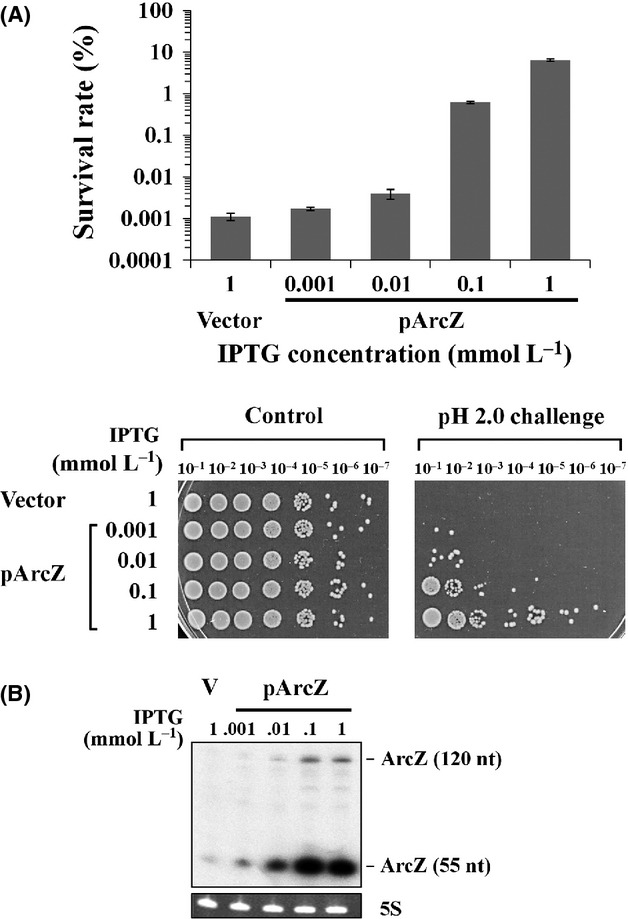
Effect of ArcZ overexpression on acid resistance of *E. coli* cells. (A) Survival rates of MG1655 transformed with IPTG-inducible RNA expression plasmid overexpressing ArcZ. Exponentially growing *Escherichia coli* cells were exposed to pH 2.0 for 1 h. Expression of ArcZ was increased with increasing IPTG concentrations. The RNA expression vector, pHMB1, was used as the control plasmid. Before and after acid exposure, cells were serially diluted in LB media and plated on LB agar plates. The survival rate was calculated based on the ratio of CFU of cells after acid exposure to CFU before acid exposure. Tenfold serial dilutions of cultures exposed to acid challenge were additionally spotted on LB agar and grown overnight. Cells containing the vector plasmid grown in the presence of 1 mmol/L were used as a control, instead of cells containing RNA expression plasmids grown at zero concentrations of IPTG. (B) IPTG-induced overexpression of ArcZ was analyzed by Northern blot analysis. A ^32^P-labeled antisense oligonucleotide for ArcZ was used as the probe.

As GadA/B induced by overexpression of ArcZ is a key enzyme in the AR2 system, we further examined the participation of ArcZ in AR of *E. coli* cells. For this purpose, we cloned the ArcZ sequence into the IPTG-inducible RNA expression vector, pHMB1, to effectively control the ArcZ expression. Exponentially growing *E. coli* cells were challenged at pH 2.0 in the presence of 1.5 mmol/L glutamate for 1 h, and their survival rates were determined. Our results showed that survival rates increase with the IPTG concentration (up to 5,000-fold at 1 mmol/L) (Fig. 2A). We also found that ArcZ levels were correlated with survival rates (Fig. 2B), supporting its contribution to acid tolerance in *E. coli* cells.

### RpoS-dependent AR2 system activation by rpoS-activating sRNAs

**Figure 3:**
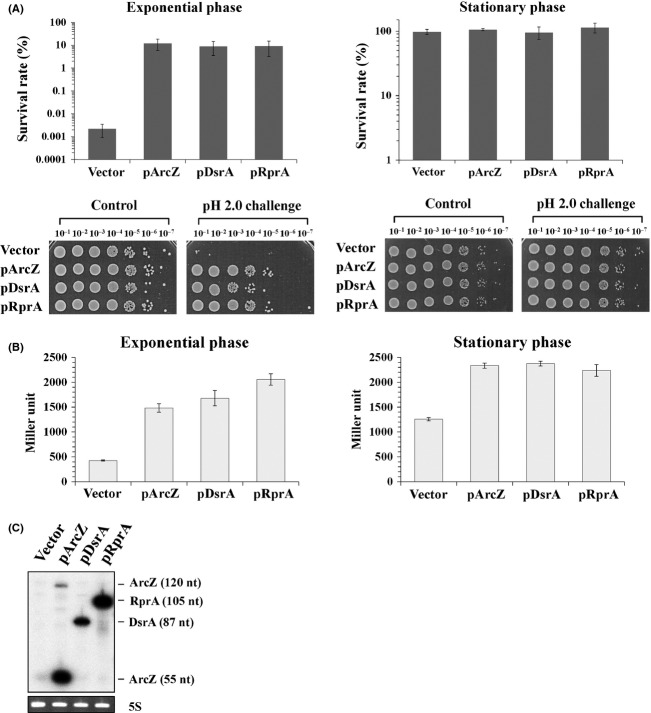
Effect of overexpression of *rpoS*-activating sRNAs on acid resistance of *Escherichia coli*. (A) Acid resistance of *E. coli* cells overexpressing DsrA, RprA, and ArcZ, respectively, was examined. Survival rates of exponentially growing or stationary-phase cells after exposure to pH 2.0 for 1 h were determined. Overexpression of sRNAs was induced by 1.0 mmol/L IPTG. Values are averaged from at least three independent experiments. Tenfold serial dilutions of cultures exposed to acid challenge were additionally spotted on LB agar and grown overnight. (B) *β*-Galactosidase activities from cells containing a *rpoS-lacZ* translational fusion (SG30013) were measured upon sRNA overexpression at the exponential or stationary phase. (C) Overexpression of *rpoS-*activating sRNAs from the corresponding RNA expression plasmids was confirmed using Northern blot analysis. Antisense oligonucleotides for ArcZ, DsrA, and RprA were mixed, labeled with ^32^P, and used as probes.

As ArcZ destabilizes antisense *arcB* mRNA and activates *rpoS* translation (Mandin and Gottesman 2010), we first tested whether enhanced AR observed with ArcZ overexpression is induced through *arcB* regulation (Fig. S1). However, upon expression of ArcZ in *arcB* mutant cells, enhanced AR was still observed, suggesting that *arcB* expression is not associated with ArcZ-induced AR. Therefore, we focused on whether ArcZ-induced translational activation of *rpoS* is responsible for AR. It was previously shown that the general stress response sigma factor RpoS plays a critical role in AR systems in commensal and pathogenic *E. coli* strains (Lin et al. 1996; Price et al. 2000). In addition to ArcZ, sRNAs such as DsrA and RprA are known to activate *rpoS* translation. Upon IPTG induction of DsrA or RprA from RNA expression plasmids, exponentially growing *E. coli* cells displayed significantly increased survival rates, similar to cells overexpressing ArcZ (Fig. 3A). Activation of *rpoS* translation by overexpression of all three sRNAs was confirmed using a *rpoS-lacZ* translational fusion (Fig. 3B). Overexpression of each sRNA led to a two-to fourfold increase in *rpoS* expression.

We further examined the effects of *rpoS-*activating sRNAs on cells grown to the stationary phase. When stationary-phase cells were challenged to pH 2.0 in the presence of 1.5 mmol/L glutamate for 1 h, their survival rates were 100%, even in the absence of sRNA overexpression, suggesting that stationary cells have already acquired sufficient tolerance to the acid challenge. However, *rpoS* expression was still increased about twofold upon overexpression of any of the three sRNAs (Fig. 3B), suggesting that stationary-phase cells do not require further RpoS for AR under these conditions.

**Figure 4:**
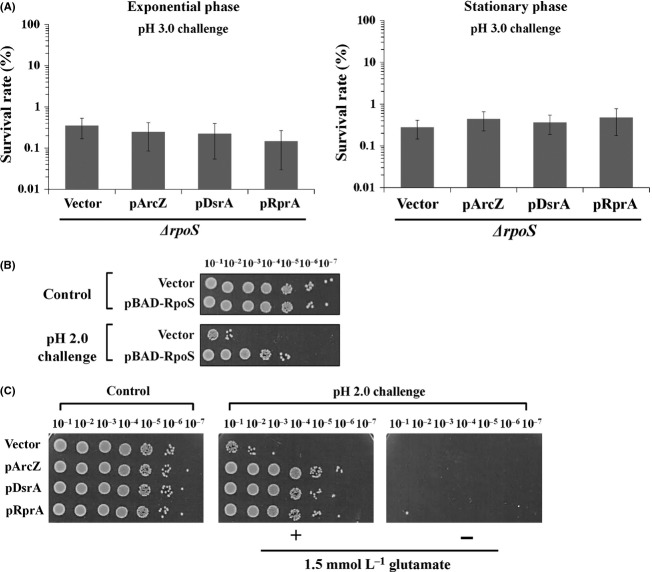
Role of RpoS in enhanced acid resistance by *rpoS-*activating sRNAs. (A) Survival rates of *ΔrpoS* mutant cells overexpressing DsrA, RprA, and ArcZ, respectively, after exposure to pH 3.0 were determined. As *ΔrpoS* cells did not survive during acid challenge at pH 2.0, cells were challenged at pH 3.0 instead. Overexpression of sRNAs was induced by 1.0 mmol/L IPTG. (B) Wild-type MG1655 cells overexpressing RpoS were exposed to pH 2.0. Overexpression of RpoS was induced from pBAD-RpoS by 0.002% arabinose in exponentially growing cells. Acid resistance was evaluated by analysis of colony-forming abilities of serial 10-fold dilutions of the cultures. (C) Glutamate dependency of acid resistance. Exponentially growing wild-type MG1655 cells were exposed to pH 2.0, and their colony-forming abilities analyzed by 10-fold serial dilution.

Next, we determined whether enhanced AR by the *rpoS*-activating sRNAs is achieved through RpoS in experiments with *rpoS* mutant cells. Unlike wild-type cells, *rpoS* mutant cells did not survive during acid challenge at pH 2.0, even with overexpression of *rpoS*-activating sRNAs, regardless of the growth phase (exponential growth or stationary) (Fig. S2). Subsequently, cells were challenged at pH 3.0 instead of pH 2.0. Notably, when *rpoS-*activating sRNAs were overexpressed, survival rates of *rpoS* mutant cells at the exponential and stationary phases were not increased (Fig. 4A), suggesting that the promotion of AR by these sRNAs is *rpoS* dependent. Moreover, overexpression of RpoS led to enhanced AR of exponentially growing *E. coli* cells (Fig. 4B), similar to that observed upon overexpression of *rpoS-*activating sRNAs. Our data clearly indicate that sRNAs confer AR in exponentially growing cells through upregulation of *rpoS*.

As *hfq* mutant cells showed extreme sensitivity to acid challenge as much as *rpoS* mutant cells did in our experimental conditions (Fig. S2) and overexpression of sRNAs in *hfq* mutant cells did not increase AR (G. Bak and Y. Lee, unpublished results), a further study on *hfq* has not been done.

As GadA/B, glutamate decarboxylases of the AR2 system, was highly accumulated upon overexpression of ArcZ, external glutamate that is an essential component of the AR2 system was included in AR assay. We examined whether AR induced by *rpoS-*activating sRNAs is increased when glutamate is omitted in acid challenge media. Enhanced AR was not observed in the absence of glutamate, indicating that *rpoS*-activating sRNAs act through the glutamate-dependent AR2 system (Fig. 4C).

### Role of GadX in promotion of AR by rpoS-activating sRNAs

**Figure 5:**
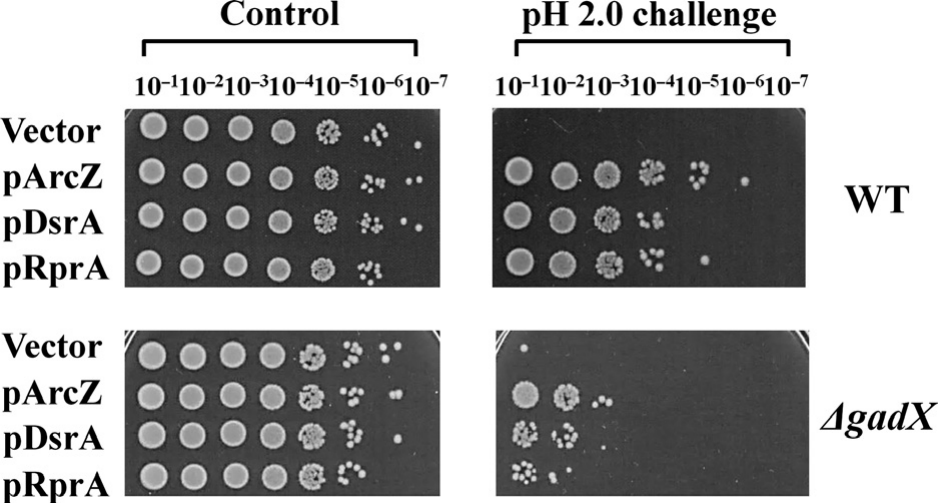
Role of GadX in enhanced acid resistance by *rpoS-*activating sRNAs. Expression of each *rpoS*-activating sRNA was induced with 1 mmol/L IPTG in *gadX* mutant cells, and exponentially growing cells exposed to pH 2.0. Acid resistance was evaluated based on the colony-forming abilities of serial 10-fold dilutions of the cultures. The results are representative of at least three independent experiments.

To identify the downstream molecules participating *rpoS-*activating sRNA-mediated AR, we assessed the involvement of GadX in this process. GadX is known to be a transcriptional activator of the *gadE* gene encoding the key transcriptional activator for *gadA* and *gadBC* genes (Masuda and Church 2003; Tramonti et al. 2006; Sayed et al. 2007; Sayed and Foster 2009). Transcription of *gadX* is not only primarily induced by RpoS in cells cultured in neutral rich media at the stationary phase, but also increased under acidic pH conditions (Ma et al. 2002, 2003b). To ascertain whether GadX is involved in the AR-acquiring process by *rpoS*-activating sRNAs, we examined the survival rates of *gadX* mutant cells overexpressing sRNAs. Upon challenge of exponentially growing cells at pH 2.0, enhanced AR was significantly decreased in the absence of *gadX* (Fig. 5), suggesting a role in enhanced AR. However, overexpressed sRNAs conferred some AR to *gadX* mutant cells, in contrast to *rpoS* mutant cells that showed no survival regardless of sRNA expression. Our findings support the existence of RpoS-dependent and GadX-independent pathways for AR enhancement by *rpoS-*activating sRNAs.

### AR in the absence of rpoS-activating sRNAs

**Figure 6:**
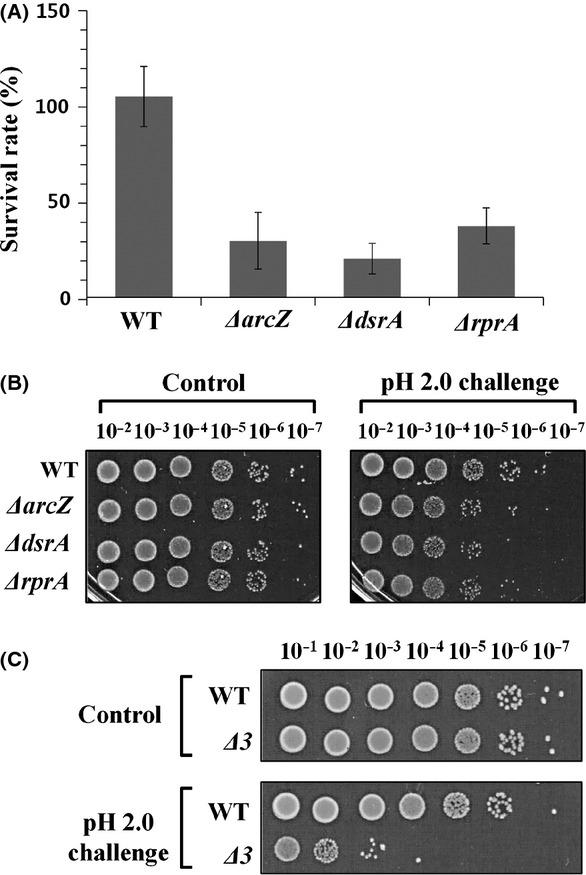
Acid resistance of cells lacking *rpoS-*activating sRNA genes. (A) Survival rates of *Escherichia coli* mutant cells with deletion of each *rpoS-*activating sRNA gene under conditions of acid shock (pH 2.0) were determined using stationary-phase cells (shown in linear scale). Values are averaged from at least three independent experiments. (B) Acid resistance of mutant cells was evaluated based on colony-forming ability. (C) Acid resistance of MG1655*Δ*3 cells lacking all three *rpoS-*activating sRNA genes was evaluated based on the colony-forming abilities of stationary-phase cells.

**Figure 7:**
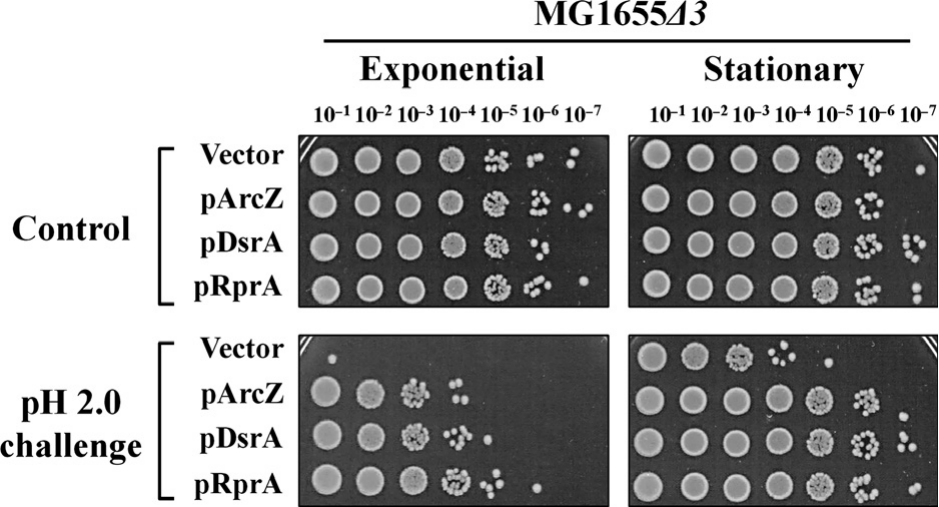
Recovery of acid resistance of mutant cells lacking all three *rpoS-*activating sRNA genes induced by single sRNAs. Each *rpoS-*activating sRNA was overexpressed in the triple mutant cells, and acid resistance evaluated based on colony-forming abilities of both exponentially growing and stationary-phase cells. Overexpression of sRNAs was induced by 1.0 mmol/L IPTG.

We examined the contribution of each *rpoS-*activating sRNA to AR using mutant strains lacking these sRNAs. Each mutant strain showed a three-to fivefold reduction in survival rates of stationary-phase cells upon acid challenge at pH 2.0 (Fig. 6A and B). When MG1655*Δ*3, a mutant strain lacking all three sRNA genes, was challenged, the survival rate was drastically reduced by about 1,000-fold (Fig. 6C). As all three *rpoS-*activating sRNAs are highly expressed in the stationary phase (Argaman et al. 2001), maintenance of the appropriate cellular levels of these sRNAs appears essential for *E. coli* cells to acquire full AR. Next, we examined the effects of overexpression of individual *rpoS-*activating sRNAs on AR defects of MG1655*Δ*3 cells. Upon overexpression of each sRNA in the MG1655*Δ*3 strain, AR at the stationary phase was fully recovered to the wild-type level and up to the level of wild-type cells overexpressing *rpoS-*activating sRNA at the exponential phase (Fig. 7), suggesting that the three sRNAs play the same role in AR.

### Induction of sRNA expression during acid challenge

**Figure 8:**
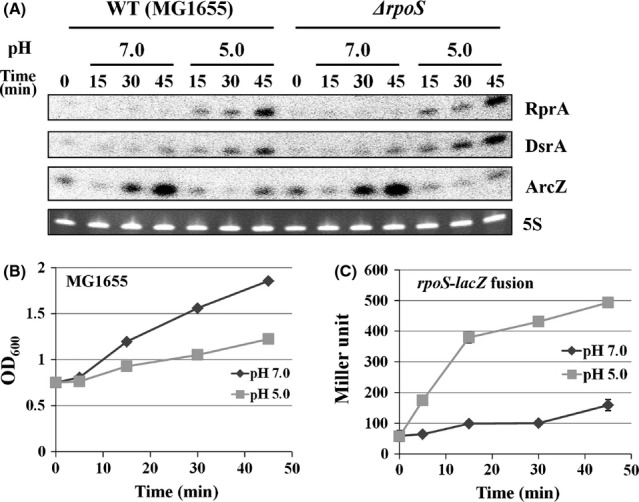
Induction of *rpoS-*activating sRNAs and activation of *rpoS* translation upon acid challenge. Overnight cultures of wild-type and *ΔrpoS* cells were diluted 1:100 in LB medium and grown for 2 h at 37°C. Cultures were split into two and either maintained or subjected to pH 5.0 (LB buffered by 100 mmol/L MES), then further incubated at 37°C. Aliquots were sampled at specific time intervals for Northern blot analysis (A), and for growth curves (B). Cells containing a *rpoS-lacZ* translational fusion (SG30013) was also shifted to pH 5.0 and aliquots were sampled for *β*-galactosidase assay (C).

All three *rpoS-*activating sRNAs are highly expressed in stationary phase, but it is not known whether they are induced during acid challenge. To examine their expression levels upon acid challenge, exponentially growing cells were subjected to moderate acid conditions of pH 5.0, as more harsh conditions may cause cell death. We observed increased expression of DsrA and RprA upon the pH shift, but not ArcZ (Fig. 8A). Expression of ArcZ was slightly reduced, probably due to the reduced growth rate because *E. coli* cells grew slowly when shifted to pH 5.0 (Fig. 8B).

As translational activation of *rpoS* upon acid exposure has been reported (Bearson et al. 1996; Battesti et al. 2011) in *Salmonella typhimurium*, we further examined whether exposure to low-pH triggers DsrA and RprA expression in *rpoS* mutant cells. DsrA and RprA were still induced in *rpoS* mutant cells (Fig. 8A). We also found that the shift of *E. coli* cells to pH 5.0 increased expression of *rpoS*-*lacZ* translational fusion (Fig. 8C), suggesting that acid-stimulated DsrA and RprA expression is responsible for increased translation of *rpoS* upon acid exposure.

## Discussion

**Figure 9:**
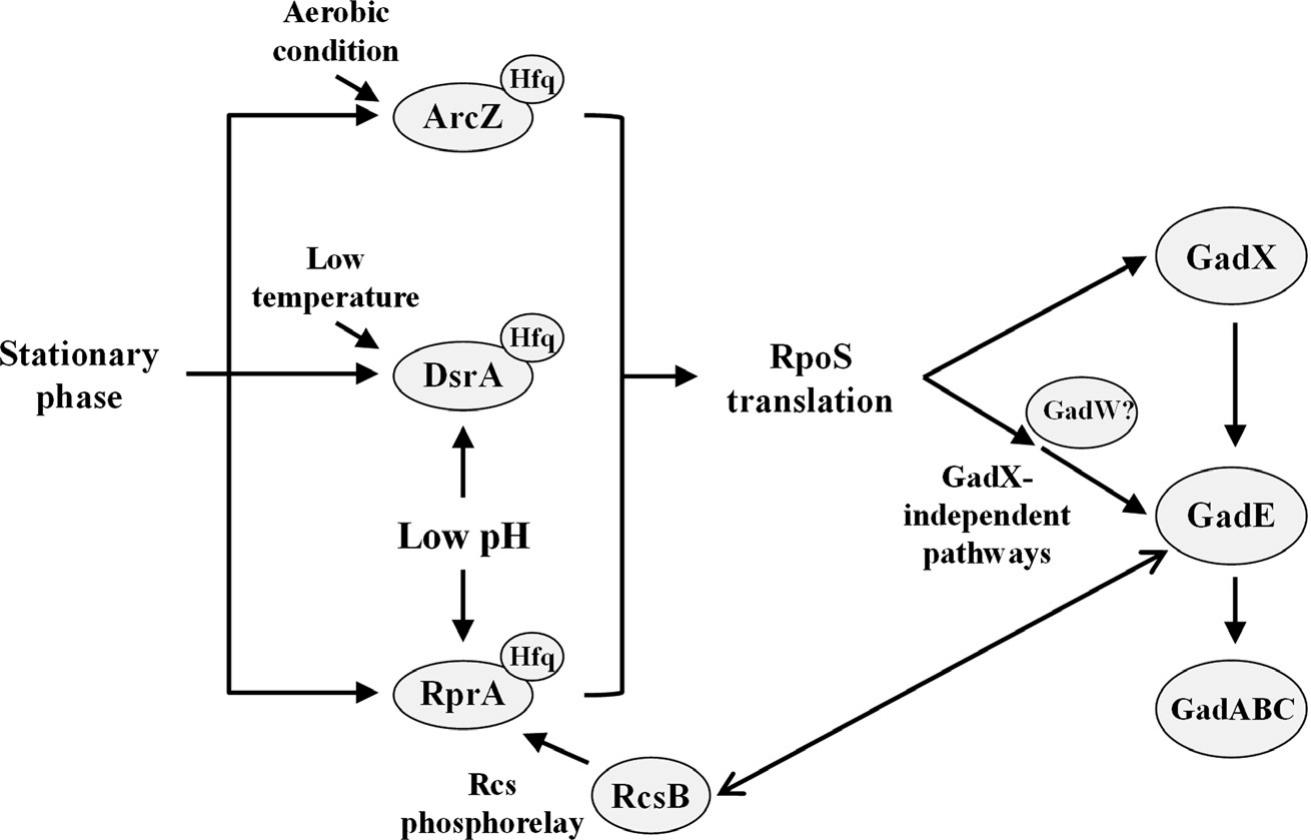
Model for acid resistance acquisition by *rpoS*-activating sRNAs in *Escherichia coli* cells. All three sRNAs are upregulated in the stationary phase. Expression of ArcZ and DsrA is induced under aerobic conditions and low temperatures, respectively. Expression of RprA is activated by Rcs phosphorelay. Expression of DsrA and RprA, but not ArcZ, is induced under acid stress. RpoS is activated by all three sRNAs in a Hfq-dependent manner. RpoS promotes *gadX* expression, and subsequently, GadX activates expression of GadE, which is a transcriptional activator for *gadA* and *gadBC* encoding key components in the AR2 system. RpoS-dependent, GadX-independent pathways of GadE induction also exist. RcsB, a response regulator of Rcs phosphorelay system, interacts with GadE to activate GadE.

In this study, three *rpoS*-activating sRNAs, ArcZ, DsrA, and RprA, once they are expressed, were shown to play an equal role in conferring AR to *E. coli* cells through the AR2 system. This sRNA-mediated AR was totally dependent on RpoS and partially on GadX. Furthermore, DsrA and RprA, but not ArcZ, were induced upon exposure to acid. On the basis of these findings, we propose a model of acid stress response by *rpoS-*activating sRNAs (Fig. 9).

RpoS is induced by variety of general stress conditions and aids in protecting cells against stress (Battesti et al. 2011). Acid shock is known to increase the translation and stability of RpoS in *S. typhimurium* and *E. coli* in LB media (Bearson et al. 1996; Heuveling et al. 2008; Battesti et al. 2011). We showed that a shift of exponentially growing *E. coli* cells to pH 5.0 not only increases the expression of translational *rpoS-lacZ* fusions but also induces DsrA and RprA. Therefore, acid stress appears to induce *rpoS* expression through induction of DsrA and RprA. ArcZ was not induced by the shift to pH 5.0, but activation of *rpoS* by ArcZ induced by other stimuli, such as aerobic conditions, can lead to AR of cells. As all three *rpoS-*activating sRNAs are abundant in the stationary phase (Argaman et al. 2001), high acid tolerance of stationary cells is mainly due to expression of RpoS induced by sRNAs. Cells depleted of any of the *rpoS-*activating sRNAs showed a three-to fivefold decreased AR at the stationary phase, suggesting that all three sRNAs are required for full AR. The three *rpoS-*activating sRNAs are triggered by different signals: ArcZ is highly expressed under aerobic conditions, DsrA is highly expressed at low temperatures and osmotic stress (Sledjeski et al. 1996; Majdalani et al. 2001), and RprA is induced by osmotic stress through Rcs phosphorelay (Majdalani et al. 2002; Majdalani and Gottesman 2005). Interestingly, RcsB protein, a response regulator of Rcs phosphorelay system, has been reported to interact with GadE to activate GadE (Castanie-Cornet et al. 2010). However, all three *rpoS-*activating sRNAs perform the same function in activating RpoS required for AR regardless of origin because overexpression of a single *rpoS-*activating sRNA can complement reduced acid tolerance caused by the absence of other sRNAs. Hfq is also essential for enhanced AR by *rpoS-*activating sRNAs (Fig. S2), possibly because sRNA-mediated activation of *rpoS* translation by these sRNAs is Hfq dependent (Battesti et al. 2011).

Among the known AR systems in *E. coli*, AR1 and AR2 require RpoS at least under certain conditions. While the mechanism underlying AR1 is not well understood, many regulators are involved in the AR2 pathway and function in different ways, depending on the growth conditions. Glutamate decarboxylases, GadA and GadB, key enzymes of the AR2 system, are induced by RpoS-dependent expression of GadX and subsequently GadX-dependent expression of GadE in stationary-phase cells grown in neutral LB media (Tramonti et al. 2002). On the other hand, exponentially growing cells have no acid tolerance at pH 2.0, possibly because GadA and GadB are not expressed during exponential phase (Ma et al. 2002; Tramonti et al. 2002). However, overexpression of *rpoS-*activating sRNAs can confer AR to exponentially growing cells through RpoS-GadX because direct overexpression of RpoS also promotes AR in *E. coli* cells. The finding that *ΔgadX* cells overexpressing sRNAs showed reduced AR, but were more acid tolerant than cells lacking RpoS supports the existence of RpoS-dependent, GadX-independent pathways for AR. Another AraC-family regulator, GadW, was reported to affect GadX-dependent activation of GadE (Ma et al. 2002, 2003b). However, in our experiments, *gadW* mutation did not affect AR promotion by sRNAs (Fig. S3), suggesting that *gadW* is not directly involved in sRNA-mediated AR. Although the RpoS-dependent, GadX-independent pathways remain to be demonstrated, they may subsequently involve activation of GadE, as overexpression of GadE leads to AR via expression of *gadA* and *gadBC* in *gadX* as well as *rpoS* mutant cells (Ma et al. 2003a). Thus, we conclude that enhanced AR by *rpoS-*activating sRNAs is conferred via a GadX-dependent or-independent RpoS-GadE circuit in both exponentially growing and stationary-phase cells.

## Supporting Information

**Figure S1.** Acid resistance of *ΔacrB* cells overexpressing ArcZ. Exponentially growing cells in the presence of 1 mmol/L IPTG were exposed to pH 2.0 for 1 h. Tenfold serial dilutions of cultures were spotted on LB agar and grown overnight. The results are representative of at least three independent experiments. **Figure S2.** Acid resistance of *Δhfq* and *ΔrpoS* cells. Exponentially growing or stationary growing *Δhfq* and *ΔrpoS* cells were exposed to pH 2.0 or 3.0 for 1 h. Acid resistance was analyzed with serial 10-fold dilutions of acidchallenged cultures. The results are representative of at least three independent experiments. **Figure S3.** Effects of GadW on acid resistance promoted by *rpoS*-activating sRNAs. Exponentially growing or *ΔgadW* cells were exposed to pH 2.0. Acid resistance was analyzed with serial 10-fold dilutions of acid-challenged cultures. Overexpression of sRNAs was induced by 1.0 mmol/L IPTG. The results are representative of at least three independent experiments. **Figure S4.** Expression of ArcZ in wild-type and *ΔarcZ* backgrounds. Total RNA samples were extracted from wild-type MG1655 and *ΔarcZ* cells. Samples were taken from indicated time points after 1:100 dilution of the overnight culture. Expression of ArcZ sRNA was analyzed by Northern blot analysis. A ^32^P-labeled oligonucleotide specific to ArcZ was used as the probe. **Table S1.** Oligonucleotide sequences used in this study.
