# Supplementary material for: Roles of rpoS-activating small RNAs in pathways leading to acid resistance of Escherichia coli
Source: Microbiologyopen. 2013 Dec 8;3(1):15–28. doi: 10.1002/mbo3.143 (PMC3937726; doi:10.1002/mbo3.143)
Supplement: Supplementary file 1 — Figure S1. Acid resistance of ΔacrB cells overexpressing ArcZ. Exponentially growing cells in the presence of 1 mmol/L IPTG were exposed to pH 2.0 for 1 h. Tenfold serial dilutions of cultures were spotted on LB agar and grown overnight. The results are representative of at least three independent experiments. Figure S2. Acid resistance of Δhfq and ΔrpoS cells. Exponentially growing or stationary growing Δhfq and ΔrpoS cells were exposed to pH 2.0 or 3.0 for 1 h. Acid resistance was analyzed with serial 10-fold dilutions of acidchallenged cultures. The results are representative of at least three independent experiments. Figure S3. Effects of GadW on acid resistance promoted by rpoS-activating sRNAs. Exponentially growing or ΔgadW cells were exposed to pH 2.0. Acid resistance was analyzed with serial 10-fold dilutions of acid-challenged cultures. Overexpression of sRNAs was induced by 1.0 mmol/L IPTG. The results are representative of at least three independent experiments. Figure S4. Expression of ArcZ in wild-type and ΔarcZ backgrounds. Total RNA samples were extracted from wild-type MG1655 and ΔarcZ cells. Samples were taken from indicated time points after 1:100 dilution of the overnight culture. Expression of ArcZ sRNA was analyzed by Northern blot analysis. A 32P-labeled oligonucleotide specific to ArcZ was used as the probe. Table S1. Oligonucleotide sequences used in this study. [file mbo30003-0015-sd1.docx]

**SUPPLEMENTARY FIGURE LEGENDS**

**Supplementary Figure 1. Acid resistance of *ΔacrB* cells overexpressing ArcZ.** Exponentially growing cells in the presence of 1 mM IPTG were exposed to pH 2.0 for 1 h. 10-fold serial dilutions of cultures were spotted on LB agar and grown overnight. The results are representative of at least three independent experiments.

**Supplementary Figure 2. Acid resistance of *Δhfq* and *ΔrpoS* cells.** Exponentially growing or stationary growing *Δhfq* and *ΔrpoS* cells were exposed to pH 2.0 or 3.0 for 1 h. Acid resistance was analyzed with serial 10-fold dilutions of acid challenged cultures. The results are representative of at least three independent experiments.

**Supplementary Figure 3. Effects of GadW on acid resistance promoted by *rpoS-*activating sRNAs.** Exponentially growing or *ΔgadW* cells were exposed to pH 2.0. Acid resistance was analyzed with serial 10-fold dilutions of acid-challenged cultures. Overexpression of sRNAs was induced by 1.0 mM IPTG. The results are representative of at least three independent experiments.

**Supplementary Figure 4. Expression of ArcZ in wild-type and *ΔarcZ* backgrounds.**

Total RNA samples were extracted from wild-type MG1655 and *ΔarcZ* cells. Samples were taken from indicated time points after 1:100 dilution of the overnight culture. Expression of ArcZ sRNA was analyzed by Northern blot analysis. A ^32^P-labeled oligonucleotide specific to ArcZ was used as the probe.

**Supplementary Table 1. Oligonucleotide sequences used in this study**

| **Name** | **Sequences (5'-3', restriction sites underlined)** | **Description** |
| --- | --- | --- |
| ArcZ NP | GGCTAGACCGGGGTGCGCGAATAC | ArcZ northern probe |
| DsrA NP | GTTACACCAGGAAATCTGATGTGTT | DsrA northern probe |
| RprA NP | GGGGATTTCCATGCTTATAAATC | RprA northern probe |
| 5S+90 | GAGACCCCACACTACCATCGG | 5S northern blot |
| BHI_SH-107 | CCGGGATCCCTTTCGCAATTGACTGAAAC | *arcZ* cloning into pGEM3 |
| SH+208_ERI | CCGGAATTCGATAACGTCGGTGAATGG | *arcZ* cloning into pGEM3 |
| ArcZER1Fw | GGCCGAATTCGTGCGGCCTGAAAAAC | *arcZ* cloning into pHMB1 |
| ArcZXb1Rv | GGCTTCTAGAGAAAGCGTGGGTGGC | *arcZ* cloning into pHMB1 |
| RprAER1Fw | GGCTGAATTCACGGTTATAAATCAA | *rprA* cloning into pHMB1 |
| RprAXb1Rv | GGCTTCTAGACGAGGTAGCGAAGCG | *rprA* cloning into pHMB1 |
| DsrAER1Fw | GGCTGAATTCAACACATCAGATTT | *dsrA* cloning into pHMB1 |
| DsrAXb1Rv | GGCTTCTAGATGAAGTGAATCGTTG | *dsrA* cloning into pHMB1 |
| ΔP10arcZP1 | CAGACCGCCTGCGCACCGCTGCGTGAAATAGCTAACAACGTCAGAAGAACTCGTCAAG | Constructing MG1655*ΔarcZ* |
| ΔP10arcZP2 | TATGGACAGCAAGCGAACCG | Constructing MG1655*ΔarcZ* |
| ΔP10arcZP3 | CGGTTCGCTTGCTGTCCATAGCATCCGCTCAGAATTACGC | Constructing MG1655*ΔarcZ* |
| ΔP10arcZP4 | ATTATTATGATGAGTTACAAGGGCACAG | Constructing MG1655*ΔarcZ* |
| Sdm P1 | TCATTTAAGTTTTGCCTCGAGAACTGCGTGCGGCCTG | Mutagenic primer for arcZ -10 region (CTCGAG) |
| Sdm P2 | CAGGCCGCACGCAGTTCTCGAGGCAAAACTTAAATGA | Mutagenic primer for arcZ -10 region (CTCGAG) |
| ΔdsrA_Fwd | GGGTGACGTGCGTCACATTTCTATTCATAAGTAGCGTTAATCATTTCCGGGGATCCGTCGACC | Constructing MG1655*ΔdsrA* |
| ΔdsrA_Rev | ATAAAAAAATCCCGACCCTGAGGGGGTCGGGATGAAACTTGCTTATGTAGGCTGGAGCTGCTTCG | Constructing MG1655*ΔdsrA* |
| ΔrprA_Fwd | CATTCAGCTGGTAGTACCTGTCGCAAATTCTTTACAGTTTTTTGTAGGCTGGAGCTGCTTCG | Constructing MG1655*ΔrprA* |
| ΔrprA_Rev | TCAGCCTGCTGACGGCTTGAAGAGAGTCACAGTATCTTGTGCAACTTCCGGGGATCCGTCGACC | Constructing MG1655*ΔrprA* |
| BamHIss6F | CGGGATCCATAAATGTGAGCGGATAACATTGACATTGTGAGCGG | Constructing pHMB1 |
| HindIIIss6R | CCCAAGCTTGTCGACTCTAGAATTATATTGTTATCCGCTCACAATGTC | Constructing pHMB1 |
| pHMB1oligo1 | AATTCGAGCTCGCGTCTAGAGCCGGCAAGCTTGGAGTACGTAAAAACCCGCTTCGGCGGGTTTTTGCTTTTGGAGGGGC | Constructing pHMB1 |
| pHMB1oligo2 | AGCTGCCCCTCCAAAAGCAAAAACCCGCCGAAGCGGGTTTTTACGTACTCCAAGCTTGCCGGCTCTAGACGCGAGCTCG | Constructing pHMB1 |


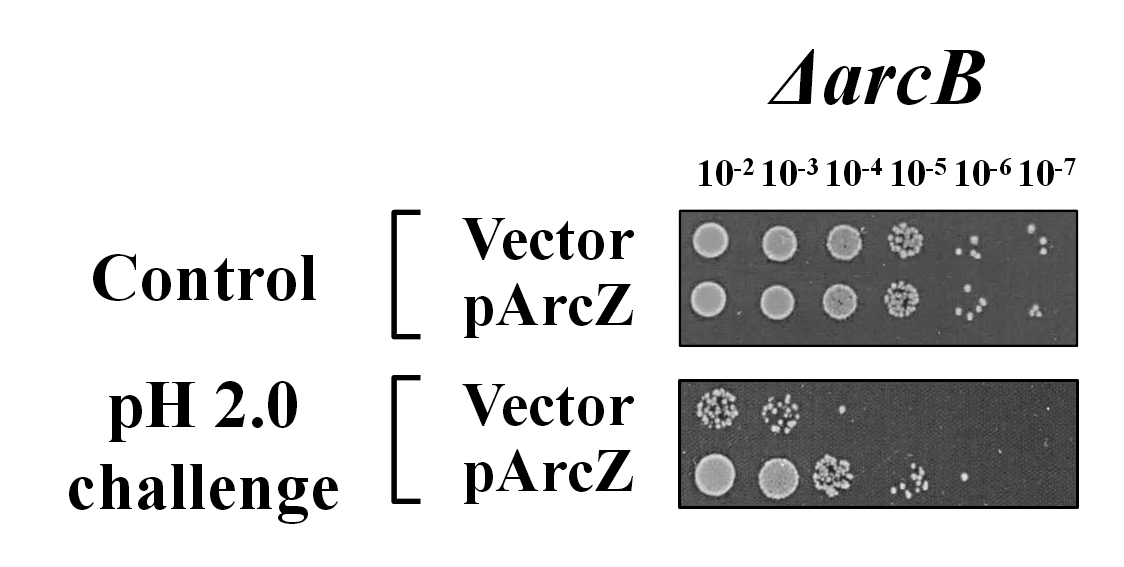


Supplementary Figure 1. Acid resistance of *ΔacrB* cells overexpressing ArcZ. (Bak et al.)





Supplementary Figure 2. Acid resistance of *Δhfq* and *ΔrpoS* cells. (Bak et al.)


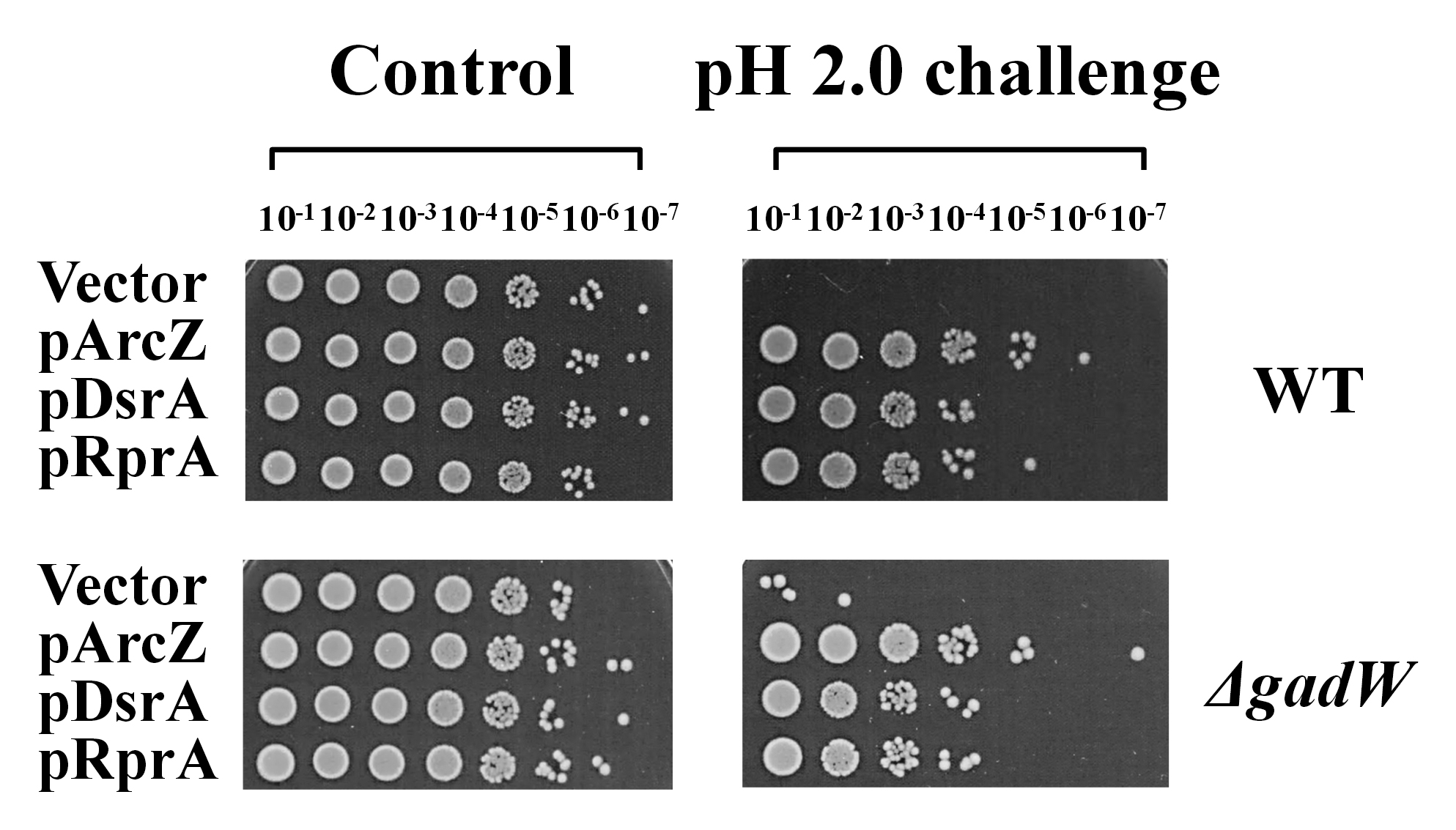


Supplementary Figure 3. Effects of GadW on acid resistance promoted by *rpoS-*activating sRNAs. (Bak et al.)

Supplementary Figure 4. Expression of ArcZ in wild-type and *ΔarcZ* backgrounds. (Bak et al.)
